# Impact of Care-Recipient Relationship Type on Quality of Life in Community-Dwelling Older Adults With Dementia Over Time

**DOI:** 10.1177/08919887231215044

**Published:** 2023-11-11

**Authors:** Aiping Lai, Lauren E Griffith, Ayse Kuspinar, Jenna-Smith Turchyn, Julie Richardson

**Affiliations:** 1 3710 School of Rehabilitation Science, McMaster University, Hamilton, ON, Canada; 2 62703 Department of Health Research Methods, Evidence, and Impact, McMaster University, Hamilton, ON, Canada

**Keywords:** quality of life, older adults, dementia, care-recipient relationship type, functional limitations

## Abstract

**Introduction:**

Maintaining quality of life (QoL) has been identified as the primary goal of care services for person living with dementia (PLWD).

**Methods:**

A secondary analysis was conducted on five rounds of the National Health and Aging Trends Study (NHATS) over 4 years. A generalized estimating equation (GEE) was used to examine the prediction of relationship type on older adults’ QoL through four domains: mental health, general health, functional limitations, and pain.

**Results:**

older adults cared for by an adult-child or multiple caregivers predicted increased risk for functional limitations after adjustment for their socio-demographic and dementia status (IRR = 1.53, CI [1.26, 1.86]; IRR = 1.36, CI [1.14, 1.61], respectively). The interaction between the relationship type and education was significant. Older adults with a high school education or below, who were cared for by an adult child, had a significantly higher risk of increasing functional limitations over 4 years compared to those cared for by a spouse/partner (contrast = .50, *P* = .01, 95% CI [.07, .93]; contrast=.52, *P* = .03, 95% CI [.03, 1.02]; respectively). Similarly, older adults with a high school education, who were cared for by multiple caregivers, also experienced a significantly higher risk of increasing functional limitations than those cared for by a spouse/partner (contrast = .44, *P* = .03, 95% CI [.02, .85]).

**Conclusion:**

Our findings provide evidence of the significant contribution of relationship type on PLWD’s QoL changes over time. They also help to prioritize resource allocation while addressing PLWD’s demands by socio-demographics such as education level.

## Background

“Dementia” is a general term for the impaired ability to remember, think, or perform daily activities.^1^ With the prolongation of the human lifespan, dementia has become a significant public health issue.^2^ In 2050, the number of people living with dementia (PLWD) globally is projected to increase by 204% from 50 million in 2018 to 152 million.^3^ An estimated 6.5 million Americans aged 65 years and older are living with Alzheimer’s disease, the most common type of dementia, and this number is expected to reach 12.7 million in 2050.^4^ Without a cure or effective treatment for these diseases, maintaining quality of life (QoL) has been identified as the primary goal of care services for PLWD.^5^

Quality of Life is defined as “an individual's perception of their position in life in the context of the culture and value systems in which they live and in relation to their goals, expectations, standards and concerns.”^6^ Quality of Life is a growing area of interest in dementia research. While several standardized QoL assessment tools have been specifically developed for PLWD^7^, such as the Quality of Life in Alzheimer Disease (QOL-AD), Alzheimer Disease-Related Quality of Life (ADRQL), Quality of Life in Late-Stage Dementia (QUALID), it is important to acknowledge that the complex and progressive nature of dementia presents challenges to comprehensive QoL measurement. Additionally, the subjective nature of QoL measurement becomes significantly more difficult when attempted in people with cognitive deficits such as in PLWD.^8^ Furthermore, while various factors impact the QoL of PLWD (e.g., socio-demographic characteristics, physical, psychological, etc.),^7,9^ consensus on what factors influence QoL most in PLWD is needed in order to develop effective interventions. Moreover, factors impacting the QoL of PLWD vary across different living settings (care institutions vs communities) and differ based on stakeholder perspectives (PLWD vs PLWD’s caregivers).^9^ For community-dwelling older adults, “informal caregivers are ‘the most important resource available for people with dementia’.^10,11^ About 61% of Canadian PLWD live in the community and receive care mainly from their informal caregivers,^12^ who may be family members, friends, or other unpaid caregivers (e.g., nonrelatives not affiliated with a caregiving institution).^13^ Given the existence of a preceding relationship between the care recipients and their caregivers, it is likely that the dyad will influence each other, including their responses to QoL and well-being, the strain they experience in the relationship, and the level of congruence and conflict about the care being provided.^11,14^

The care-recipient relationship type (i.e., the type of relationship/degree of kinship between caregivers and the care recipients, referred to from here on as “type of relationship”) is known to associate with QoL in informal caregivers of PLWD.^14-19^ Compared to caregivers, evidence about the impact of relationship factors on the care recipients or PLWD is limited.^20^ Existing information indicates that type of relationship influences the level of functional abilities in PLWD.^14^ Care recipients cared for by adult-child caregivers had a higher risk of experiencing functional limitations than those cared by spousal caregivers.^14^ However, the cross-sectional analysis does not allow for determining the temporal basis of relationships and limits the ability to make causal conclusions.^21^ In addition, previous studies have found that PLWD’s QoL was influenced not only by their severity of dementia^22,23^ but also by their socio-demographics (e.g., age, race, living arrangements).^24,25^ Therefore, when evaluating the effects of type of relationship, it is also important to consider the potential impact of PLWD’s dementia condition and socio-characteristics.

Given the absence of existing studies regarding the impact of the relationship type on PLWD’s QoL and limitations in establishing causal relationships due to the nature of cross-sectional studies, longitudinal data-derived evidence is important to provide insights to healthcare professionals and caregivers. Therefore, we used five rounds of National Health and Aging Trends Study (NHATS) data (Round five to nine) to address these gaps. Specifically, we aim to address two questions:

(1) Does type of relationship or caregiving being shared predict a change in PLWD’s QoL over four years after adjusting for socio-demographics and dementia status?
(2) Does the effect of type of relationship or caregiving being shared differ by socio-demographics and dementia status?

## Methods

This was a longitudinal secondary analysis study.

### Data Sources and Participants Selected

The present study used de-identified data from the NHATS Round five in 2015 through Round nine in 2019. As a population-based in-person survey that measures late-life disability from a nationally representative sample of Medicare beneficiaries age 65 and older in the United States,^26^ the NHATS offers large sample sizes and has a comprehensive, validated disability protocol that is administered annually. NHATS is sponsored by the National Institute on Aging (grant number NIA U01AG32947) and is conducted by Johns Hopkins University. The content of the NHATS was guided by a conceptual framework that blends the International Classification of Functioning, Disability and Health (ICF) with the Nagi model of disablement.^27^ The NHATS participants were initially sampled in Round one in 2011 and replenished in Round five in 2015. Thus, using the NHATS Round five allows us to have a sample of the 2011/2015 cohort. When the older adult could not respond, the NHATS interviewed proxy respondents and collected information on reasons for using a proxy, the relationship of the proxy to the older adult, and proxy familiarity with the older adults’ daily routine.^26^

We included older adults who live in the community and receive help with certain Activities of Daily Living (ADL)- getting around inside home/building, getting out of bed, eating, bathing/showering/washing up, getting to or using the toilet, dressing-from their informal caregivers at the time of enrollment. Informal caregivers in this study refers to “family and unpaid caregivers”, who assisted a potential eligible participant with any ADLs and were either (1) related to the older adult whether paid or not, or (2) unrelated to the older adult and not paid to help.^28^ Of 8334 older adults in the original NHATS Round five dataset, 1230 participants were identified as the eligible analytical sample in the current study. If a participant did not respond in one of the follow-up rounds, no attempt was made to contact those again in the next round.

### Measures

#### Quality of Life Outcomes

We chose the ICF framework in selecting appropriate QoL measures as well as identifying determinants of QoL in this study. Previous evidence showed that all factors included in the ICF framework potentially affect an individual’s QoL and contribute to changes in their QoL over time.^29^ Guided by the ICF framework, older adults’ QoL in this study was assessed in four domains: mental health, general health, functional limitations, and pain. A recent systematic review showed that mental health, functional limitation, and pain are essential factors associated with PLWD’s QoL.^7^ In our study, mental health was presented using the Patient Health Questionnaire for Depression and Anxiety (PHQ-4), a scale with a brief screening tool for depression and anxiety symptoms that is composed of two subscales-a depression subscale from the 2-item Patient Health Questionnaire (PHQ-2) and an anxiety subscale from the 2-item Generalized Anxiety Disorder scale (GAD-2).^30^ The depression subscale of PHQ-4 measures how often the participant “had little interest or pleasure in doing things” and “felt down, depressed, or hopeless” over the past month. The anxiety subscale of PHQ-4 measures how often the participant “felt nervous, anxious, or on edge” and “was unable to stop or control worrying” over the past month. Responses to each question were recorded on a 4-point scale (scored 0-3), and the total score of the four items ranged from 0 to 12, with a higher score representing more depressive/anxiety symptoms.^26^

According to previously validated criteria, PHQ-4 can be categorized into low (0-2), mild (3-5), moderate (6-8), and severe symptoms (9-12).^30^ However, considering the small number of participants in mild and moderate categories, we created a dichotomous indicator to categorize participants into two groups using a cutoff score of 3: low (0-2) and symptomized (3-12). General health was self-rated on a 5-point scale from excellent (0) to poor (4). Pain was evaluated by asking whether or not the participants were bothered by pain in the past month and scored as yes (1) or no (0). Functional limitations were presented as the total number of activities of daily living (ADLs) that the participant received help with within the past month and scored 0 to 6 with a higher score representing more severe limitations^26^ (see Supplementary Appendix I).

#### Care-Recipient Relationship Type

The term “Care-recipient relationship” in this study represents the type of relationship between PLWD and their informal caregivers. An informal caregiver “includes any person, such as a family member, friend or neighbour, who is giving regular, ongoing assistance to another person without payment for the care given.”^31^ The type of relationship categorized four groups: (1) care from a spouse/partner; (2) care from an adult child; (3) care from an informal caregiver other than spouse/partner and adult child, such as child-in-law, sibling, friend, etc. (referred to from here on as “other caregivers”); (4) If NHATs care recipients indicated having multiple helpers/caregivers, they were assigned to the group of “multiple caregivers”, as opposed to those with a single caregiver.

#### Dementia Status

To classify older adults’ dementia status, a three-category dementia classification (probable dementia, possible dementia, and no dementia) was used, generated from the NHATS Round five (2015). For a non-proxy participant, cognitive function was assessed using a battery of cognitive tests that evaluated memory (immediate and delayed 10-word recall), orientation (date, month, year, and day of the week; naming the President and Vice President), and executive function (clock drawing test).^32^ For proxy informants, cognitive function was assessed using the AD8 Dementia Screening Interview which assesses memory, temporal orientation, judgment, and function.^32–34^ As per a previously developed and validated approach,^32^ the participant was classified into the probable dementia group if there was a self or proxy report of physician diagnosis of dementia or Alzheimer’s disease; or AD8 score ≥2; or at least two domains of cognitive tests met their respective cut points. If one domain of cognitive tests met cut point with no physician diagnosis of dementia or Alzheimer’s disease, the participant was classified into the possible dementia group. Findings from a sensitivity and specificity analysis, conducted against a clinically evaluated sample in 2010 (Aging, Demographics, and Memory Study, ADAMS, Wave E),^35^ demonstrated that the NHATS three-category dementia classification exhibited high sensitivity (85.7%) against ADAMS dementia diagnosis. Furthermore, it revealed reasonable good sensitivity (71.8%) against diagnoses of dementia or cognitive impairment not dementia (CIND), along with high specificity (83.7%) for persons classified as normal in ADAMS.^32^

### Socio-Demographics

Older adults’ socio-demographic characteristics assessed at Round five were used in analyses: age range (65-69 years, 70-74 years, 75-79 years, 80-84 years, 85-89years, ≥90 years), sex (male, female), race/ethnicity (non-Hispanic white, non-Hispanic black, Hispanic, other), annual income in quartiles (<1st quartile, 1st-2nd quartiles, 2nd- 3rd quartiles, >3rd quartile), education (below high school, high school, above high school and below bachelor’s degree, bachelor’s degree or above), marital status (married or living with a partner, unmarried including separated/divorced/widowed/never married), and living arrangements (alone, with spouse/partner only, with spouse/partner and others, with others only). The associations between these socio-demographic factors and QoL outcomes have been reported in various studies^14,24,25^

### Statistical Analyses

Categorical variables of type of relationship, dementia status, socio-demographic characteristics, and QoL subscales (pain, general health, PHQ4, functional limitations) were described using counts and percentages. Descriptive statistics were used to assess the changes in QoL subscales across five rounds (2015 to 2019). Baseline QoL subscales were examined by type of relationship using Chi-square tests for categorical variables (PHQ4, pain) and Kruskal–Wallis for ordinal (general health) and count variable (functional limitations). We used Bonferroni correction for multiple comparisons. The baseline variables of non-respondents through four years (Round six to Round nine) were compared to the included participants in Round five using Chi-square tests for categorical variables and Kruskal–Wallis for ordinal and count variables.

The generalized estimating equation (GEE) approach was used to compare the odds of participants in the four groups by the relationship types. The comparisons were over the four years across Round five-Round nine. The GEE approach takes into account the correlation of repeated measures within the same individual over the years and provides flexibility to retain the full sample of respondents (e.g., respondents with two or three consecutive time points of data can be included in the GEE analysis, while controlling for time point of administration).^36^ With a logit link function for binomial variables (PHQ4, pain), a log link function for the ordered variable (general health) and count variable (functional limitations), we built models to estimate odds ratios (OR)/incident rate ratio (IRR) and 95% confidence intervals (CI), with the group receiving care from a spouse/partner serving as the reference category. Two models were built with sequential adjustment for covariates: Model one adjusted for socio-demographic characteristics; Model two additionally controlled for dementia status. To disentangle the effects of potential interactions, we further tested for interactions between type of relationship with age, gender, marital status, dementia status, and education. Using Model two as a base model, each interaction term was tested in a separate regression model (i.e., Models three-seven). If a significant interaction term was found, a Sidak post-hoc comparison correction was then conducted to explore where the difference existed. All data were analyzed using Stata 16.0, and a two-tailed significance test with an alpha of 0.05 was set.

## Results

### Sample Characteristics

**Figure 1.**
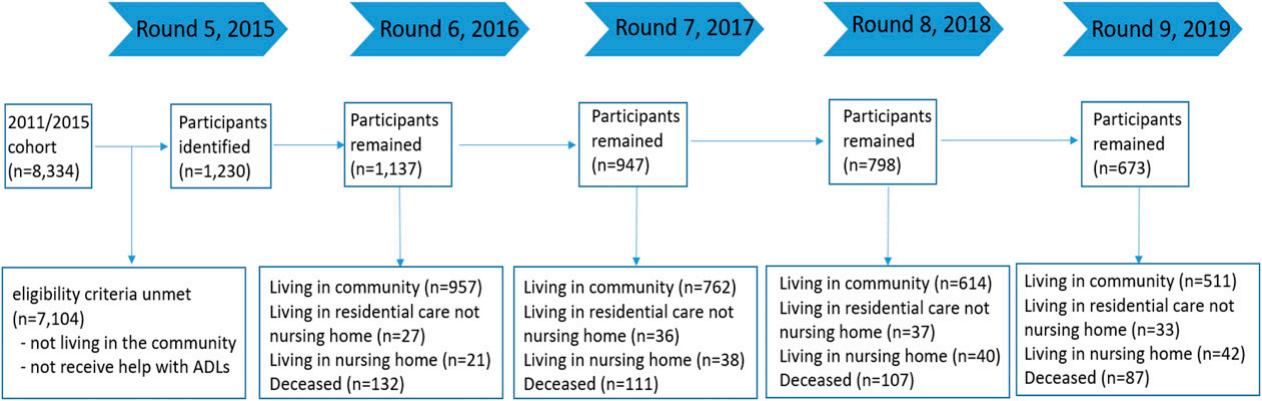
Participants over 4 years period (from round five to round nine). Note: In 2015 Round five there were 1230 participants identified. In the following rounds, some were no longer living in community; some passed away or non-response. So the number of eligible participants who remained in follow-up rounds gradually decreased. In round nine, 511 participants can be used for analysis, which is less than 50% of participant included in the Round five.

There were 1230 older adults in the analysis at Round five (see Supplementary Appendix II). The majority were female (67.1%), in an age range of 80-84 years (22.1%), non-Hispanic White (60.2%), unmarried (56.8%), living with others only (37.3%), and identified as living with no dementia (55.2%). Approximately 27.1% of the participants possessed an annual income within the 2nd-3rd quartiles ($13,000 - $22,000), and 29.5% of the participants held a high school education. There were no significant differences in socio-demographic distribution and dementia status across the five rounds. The final retention rate for participants was 92.4%, 77.0%, 64.9%, and 54.7% for Round 6, 7, 8, and 9, respectively. Reasons of missing data include non-responses, moving to institutionalized settings, and deceased (see Figure 1). Compared to the overall baseline sample, non-respondents were generally younger and had a larger percentage of people living without dementia (see Supplementary Appendix III). Other variables (e.g. race, gender) were not found to be significant between the groups. The rate of proxy respondents included in this study was 17.4% in the baseline (NHATS Round five).

**Figure 2.**
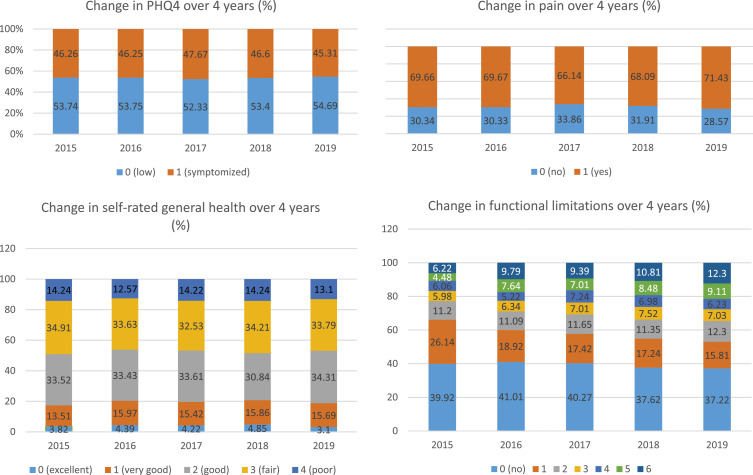
Changes in each QoL subscale cross five rounds (Round five, 2015 to Round nine, 2019). Note: PHQ4 Patient Health Questionnaire for Depression and Anxiety. PHQ-4 was categorized into two groups: low (0-2) and symptomized (3-12); General health was self-rated on a 5-point scale: excellent (0), very good (1), good (2), fair (3), and poor (4). Pain was evaluated by asking whether or not the participants were bothered by pain in the past month and scored as yes (1) or no (0); Functional limitations were presented as the total number of activities of daily living (ADLs) that the participant received help with within the past month and scored 0 to 6 with a higher score representing more severe limitations. The figure indicates that older adults had more complaints of pain and tended to receive help with more ADLs over time, especially those receiving help with five or six ADLs in Round five.

Tables 1 and 2 show the baseline distribution of participants by care-recipients relationship types, as well as the socio-demographics, dementia status, and QoL outcomes. In Round five, 24.1% of older adults received care from a spouse/partner, 28.6% received care from an adult child, 9.3% from “other”, and 38.0% from multiple caregivers. No significant differences were observed in the baseline QoL outcomes, including pain, general health, PHQ4 scores, and functional limitations, across the four groups. In general, older adults had more complaints of pain and tended to receive help with more ADLs over time, especially those receiving help with five or six ADLs in Round five (see Figure 2, Supplementary Appendix IV).

**Table 1.** Baseline Socio-Demographics and Dementia Status by the Type of Relationship.

Variables Count (percentage)	Total (n = 1230)	Type of Relationship			
		By Spouse/Partner	By Adult Child	By “Other”	By Multiples
		296 (24.1)	352 (28.6)	114 (9.3)	468 (38.0)
Sex					
Male	405 (32.9)	172 (58.1)	58 (16.5)	38 (33.3)	137 (29.3)
Female	825 (67.1)	124 (41.9)	294 (83.5)	76 (66.7)	331 (70.7)
Age					
65 to 69 years	84 (6.8)	45 (15.2)	17 (4.8)	5 (4.4)	17 (3.6)
70 to 74 years	174 (14.2)	56 (18.9)	35 (9.9)	23 (20.2)	60 (12.8)
75 to 79 years	222 (18.1)	76 (25.7)	56 (15.9)	18 (15.8)	72 (15.4)
80 to 84 years	272 (22.1)	67 (22.6)	69 (19.6)	26 (22.8)	110 (23.5)
85 to 89 years	256 (20.8)	36 (12.2)	80 (22.7)	24 (21.1)	116 (24.8)
90 + years	222 (18.1)	16 (5.4)	95 (27.0)	18 (15.8)	93 (19.9)
Race/ethnicity					
Non- hispanic white	735 (60.2)	230 (78.0)	185 (53.0)	54 (48.2)	266 (57.1)
Non- hispanic black	386 (31.6)	50 (17.0)	124 (35.5)	47 (42)	165 (35.4)
Hispanic	62 (5.1)	7 (2.4)	25 (7.2)	7 (6.3)	23 (4.9)
Other	39 (3.2)	8 (2.7)	15 (4.3)	4 (3.6)	12 (2.6)
Annual income					
<1st quartile	305 (24.8)	15 (5.1)	128 (36.4)	46 (40.4)	116 (24.8)
1st-2nd quartiles	309 (25.1)	33 (11.2)	109 (31.0)	33 (29.0)	134 (28.6)
2nd-3rd quartiles	333 (27.1)	105 (35.5)	80 (22.7)	21 (18.4)	127 (27.1)
>3rd quartile	283 (23.0)	143 (48.3)	35 (9.9)	14 (12.3)	91 (19.4)
Education					
Below high school	412 (33.8)	62 (21.0)	130 (37.1)	44 (39.6)	176 (38.0)
High school	360 (29.5)	81 (27.4)	113 (32.3)	30 (27.0)	136 (29.4)
Above high school below bachelor	271 (22.2)	88 (29.7)	69 (19.7)	26 (23.4)	88 (19.0)
Bachelor and above	177 (14.5)	65 (22.0)	38 (10.9)	11 (9.9)	63 (13.6)
Marital status					
Married/living with a partner	532 (43.3)	296 (100)	47 (13.4)	17 (14.9)	172 (36.8)
Unmarried	698 (56.8)	N/A	305 (86.7)	97 (85.1)	296 (63.3)
Living arrangements					
Alone	249 (20.2)	N/A	111 (31.5)	44 (38.6)	94 (20.1)
With spouse/partner only	376 (30.6)	248 (83.78)	18 (5.1)	12 (10.5)	98 (20.9)
With spouse/partner & others	146 (11.9)	48 (16.22)	24 (6.8)	3 (2.6)	71 (15.2)
With others only	459 (37.3)	N/A	199 (56.5)	55 (48.3)	205 (43.8)
Dementia status					
Probable dementia	371 (30.2)	59 (20.0)	127 (36.1)	34 (29.8)	151 (32.3)
Possible dementia	179 (14.6)	34 (11.5)	55 (15.6)	23 (20.2)	67 (14.4)
No dementia	678 (55.2)	202 (68.4)	170 (48.3)	57 (50.0)	249 (53.3)

yrs years; unmarried single/widowed/separated/divorced.

**Table 2.** Baseline QoL Outcomes by the Type Of Relationship.

Variables Count (Percentage)	Total (n = 1230)	Type of Relationship			
		By Spouse/Partner	By Adult Child	By “Other”	By Multiples
		296 (24.1)	352 (28.6)	114 (9.3)	468 (38.0)
Pain					
Reporting pain	854 (69.4)	212 (71.6)	248 (70.7)	74 (64.9)	320 (68.8)
			chi2 (3) = 2.0754, *P* = .557		
PHQ4					
Symptomized (PHQ4>2, ≤12)	553 (45.0)	117 (40.6)	159 (46.4)	59 (52.7)	221 (484.1)
			chi2 (3) = 6.1942, *P* = .103		
General health					
Excellent	47 (3.8)	12 (4.1)	19 (5.4)	2 (1.8)	14 (3.0)
Very good	166 (13.5)	46 (15.5)	41 (11.7)	12 (10.5)	67 (14.3)
Good	412 (33.5)	99 (33.4)	117 (33.3)	35 (30.7)	161 (34.4)
Fair	429 (34.9)	99 (33.4)	123 (35.0)	46 (40.4)	161 (34.4)
Poor	175 (14.2)	40 (13.5)	51 (14.5)	19 (16.7)	65 (13.9)
			chi2 with ties (3) = 4.154, *P* = .2453		
Functional limitations					
Not receiving help with ADL	481 (39.1)	103 (35.2)	138 (40.4)	56 (51.4)	184 (39.9)
Receiving help with 1 ADL	315 (25.6)	111 (37.9)	67 (19.6)	21 (19.3)	116 (25.2)
Receiving help with 2 ADLs	135 (11.0)	38 (13.0)	35 (10.2)	12 (11.0)	50 (10.8)
Receiving help with 3 ADLs	72 (5.9)	13 (4.4)	25 (7.3)	2 (1.8)	32 (6.9)
Receiving help with 4 ADLs	73 (5.9)	9 (3.1)	29 (8.5)	6 (5.5)	29 (6.3)
Receiving help with 5 ADLs	54 (4.4)	9 (3.1)	23 (6.7)	4 (3.7)	18 (3.9)
Receiving help with 6 ADLs	75 (6.1)	10 (3.4)	25 (7.3)	8 (7.3)	32 (6.9)
			chi2 with ties (3) = 6.362, *P* = .0953		

*yrs* years; *unmarried* single/widowed/separated/divorced; *PHQ4* Patient Health Questionnaire for Depression and Anxiety; *ADL* activity of daily living.

### Impact of Care-Recipient Relationship Type on Quality of Life Outcomes Over Time

GEE analyses indicated that older adults cared for by an adult-child or multiple caregivers predicted increased risk for functional limitations (IRR = 1.58, CI [1.35, 1.85]; IRR = 1.40, CI [1.21, 1.63], respectively), and the prediction maintains after adjustment for socio-demographic characteristics (IRR = 1.59, CI [1.30, 1.95]; IRR = 1.36, CI [1.14, 1.63], respectively). After additional adjustments for baseline dementia status, the significance was maintained for these two groups (IRR = 1.53, CI [1.26, 1.86]; IRR = 1.36, CI [1.14, 1.61], respectively). There was no statistically significant relationship between the type of care-recipient relationship and other QoL outcomes, including pain, general health, and PHQ4 scores (see Table 3).

**Table 3.** Results from GEE Models of Care-Recipient Relationship Type Prediction On QoL Outcomes Over 4 years (2015-2019).

	Model Without Adjustment for Socio-Demographics & Dementia Status				Model 1				Model 2			
	OR	*P*	95% CI		OR	*P*	95% CI		OR	*P*	95% CI	
PHQ4												
By adult child	1.18	.19	.92	1.52	.95	.77	.66	1.37	.93	.69	.64	1.34
By “others"	1.02	.93	.71	1.46	.82	.39	.53	1.28	.81	.34	.52	1.26
By multiples	1.15	.25	.91	1.47	.98	.92	.72	1.35	.99	.94	.72	1.36
Pain												
By adult child	1.07	.62	.82	1.41	.91	.62	.62	1.33	.92	.68	0.63	1.35
By “others”	1.09	.68	.73	1.61	.82	.41	.50	1.32	.83	.45	.52	1.34
By multiples	1.01	.95	.78	1.30	.90	.55	.65	1.26	.90	.53	.65	1.25
	**IRR**	***P***	**95% CI**		**IRR**	***P***	**95% CI**		**IRR**	***P***	**95% CI**	
General health												
By adult child	1.03	.41	.96	1.09	1.05	0.20	.97	1.14	1.05	.20	.97	1.14
By “others"	1.05	.26	.97	1.14	1.03	.50	.94	1.14	1.03	.50	.94	1.14
By multiples	1.01	.82	.95	1.07	1.02	.60	.95	1.09	1.02	.60	.95	1.09
Functional limitations												
By adult child	1.58	**< .01**	1.35	1.85	1.59	**<.01**	1.30	1.95	1.53	**<.01**	1.26	1.86
By “others”	1.10	.48	.85	1.43	1.25	.12	.95	1.64	1.21	.16	0.93	1.57
By multiples	1.40	**<.01**	1.21	1.63	1.36	**<.01**	1.14	1.63	1.36	**<.01**	1.14	1.61

P-values in bold indicate significant < 0.05. All models using “care by a spouse/partner” as a reference group.

*Model 1* Generalized estimating equation (GEE) model with a logit link function for PHQ4 and pain, a log link function for general health and functional limitations, and adjusted for.

socio-demographic characteristics including age, gender, race, income, education, marital status and living arrangements; *Model 2* additionally controlled for dementia status. Both models use group receiving care from a spouse/partner as the reference category; *OR* odds ratios; *IRR* incident rate ratio; *95% CI* 95% confidence intervals.

No significant interaction effects were found between the type of relationship with any of the following: age, gender, marital status, or dementia status. However, the interaction term between the type of relationship and educational attainment was significant, indicating that the effect of relationship type on functional limitations is not uniform across education level. A pairwise comparison of changes using the Sidak post hoc test revealed that for older adults with a high school education or below, those cared for by an adult child experienced a significantly higher risk of increasing functional limitations over four years, compared to those cared for by a spouse/partner (contrast = .50, *P* = .01, 95% CI [.07, .93]; contrast = .52, *P* = .03, 95% CI [.03, 1.02]; respectively). Similarly, older adults with a high school education cared for by multiple caregivers experienced significantly more risk of increasing functional limitations than those cared for by a spouse/partner (contrast=.44, *P* = .03, 95% CI [.02, .85]). See Table 4.

**Table 4.** Sidak Post-Hoc Results of Functional Limitations Differences Among the Type of Relationship for 4-Levels Education.

	Contrast	Std. Err	*P*	95% CI	
Relationship type @education					
(By adult-child vs by spouse/partner) 1	.52	.17	**.03**	.03	1.02
(By adult-child vs by spouse/partner) 2	.50	.15	**.01**	.07	.93
(By adult-child vs by spouse/partner) 3	.35	.16	.29	−.10	.79
(By adult-child vs by spouse/partner) 4	.47	.18	.12	−.05	1.00
(By others vs by spouse/partner) 1	.43	.21	.39	−.17	1.03
(By others vs by spouse/partner) 2	.06	.26	1.00	−.67	.79
(By others vs by spouse/partner) 3	−.09	.28	1.00	−.90	.72
(By others vs by spouse/partner) 4	.37	.28	.92	−.44	1.18
(By multiples vs by spouse/partner) 1	.47	.17	.06	−.01	.94
(By multiples vs by spouse/partner) 2	.44	.14	**.03**	.02	.85
(By multiples vs by spouse/partner) 3	.05	.14	1.00	−.35	.46
(By multiples vs by spouse/partner) 4	.26	.18	.84	−.24	.76

*1*: below high school; *2*: high school; *3*: above high school below Bachelor; *4*: Bachelor and above. P-values in bold indicate significant < 0.05.

## Discussion

The pool of informal caregivers for PLWD has been expanding due to an increase in dementia prevalence and a shift in the traditional family structure from a gradual decline in marriage rates.^37^ Despite this observation, impact of care-recipient relationship types on the QoL of care of recipients is nevertheless relatively unexplored.^14^ To our knowledge, this is the first longitudinal study examining the effects of the type of relationship on changes in QoL over time in PLWD. After controlling for socio-demographics and dementia status, we found that the type of relationship was associated with changes in the care-recipient’s functional limitations, one of the QoL outcomes measured in this study. There were no significant differences in QoL outcomes at Round five by the type of relationship, including functional limitations. However, older adults cared for by an adult-child or multiple caregivers predicted an increased risk for functional limitations over a four-year period, compared to those cared for by a spouse/partner. Our findings suggest that the care-recipient relationship type predicts QoL changes in PLWD, which is consistent with our previous cross-sectional study^14^ and further validates the causal relationship between the type of relationship and QoL of PLWD.

Several factors could contribute to the increased risk of functional limitations among PLWD cared for by an adult-child or multiple caregivers in comparison to those cared for by a spouse/partner: (1) Lack of consistency and attention in the complex care: Due to complexity of dementia care, PLWD often benefits from routine and consistent interactions.^38,39^ Spouse/partner caregivers may provide a more stable and continuous caregiving environment, promoting a sense of familiarity and predictability that can support functional well-being. In the contrast, adult-child or multiple caregivers may face challenges in coordinating and sharing responsibilities. Adult-child caregivers often juggle caregiving responsibilities alongside other commitments such as work and family obligations. This was exemplified in a study where spouses reported significantly less burden than adult children in relation to the direct impact of caregiving on their lives.^40^ When caregiving role was shared by different caregivers, each caregiver may adopt varying approaches and techniques in managing the needs of PLWD’s ADLs/IADLs. These may lead to a potential gaps in providing consistent support and inadequate attention to PLWD’s specific needs, which can contribute to a decline in functional limitation over time. (2) Possible learned helplessness: A previous study reported that PLWD might experience learned helplessness, a psychological state when someone has learned over time that their actions are ineffective and stop trying to do something for themselves because someone else intervenes and acts in their place.^41^ Spousal caregivers may try to sustain or reconstruct couple hood by letting their partner continue with social and household chores and try to maintain former rituals and routines.^42,43^ By comparison, interventions such as care tasks taken by an adult-child or shared among multiple caregivers may erode care recipients’ self-confidence and discourage them from engaging in daily activities, which in turn can foster a decline in their physical functioning.^42,43^ Though no significant differences were observed across the four types in terms of functional limitations in round five, adult-child and multiple caregivers exhibit a higher percentage of assistance in helping older adults with additional ADLs when compared to spousal caregivers (see Table 2). However, it's important to mention that an increase in depressive symptomatology, a key indicator of learned helplessness, was not detected in this study. Therefore, this aspect warrants further exploration through additional research endeavors.

There are no similar longitudinal studies with which we can compare our results, but previous studies reported that non-spousal caregivers had a greater desire or incidence of institutionalizing the care recipients.^44-46^ People with adult-child caregivers were more likely to be admitted into nursing homes compared to those cared for by spousal caregivers,^45,46^ and the reported reasons for nursing home placement were more related to care recipients’ condition.^47^ Although multiple factors are associated with nursing home admissions, activity limitations have been found to be strongly associated with future nursing home admission.^48^ Moreover, indicators of functional limitations were among the strongest predictors in a meta-analysis review of predicting nursing home admission among older adults in the U. S.^49^ Reinforcing this trend, a systematic review focused on predicting institutionalization revealed that 96% of the included studies underscored a significantly positive impact of functional impairment on the likelihood of being institutionalized.^50^ Hajek et al.^51^ expanded these findings by exploring the longitudinal predictors of institutionalization, highlighting the pivotal role of functional impairments in ADLs/IADLs in predicting the eventual need for institutional care.

While previous studies reported the association between depressive symptoms and functional limitations,^52,53^ along with significant differences in functional limitation changes among PLWD cared for by different caregiver types in this study, notable distinctions in PHQ4 changes across caregiver types, as well as in other QoL outcomes-pain and general health, were not found. This suggests that while the caregiver approach to assisting with ADLs may vary across caregiver types, the impact of their care on PLWD’s emotions may exhibit similarities. In addition, the sensitivity of the measurement of each QoL outcome may play a role in these observations. Functional limitations were determined by simply counting the number of ADLs being assisted, whereas the other three outcomes were assessed through responses to a series of scaled questions. It is possible that within the context of dementia, there occurs a process of adapting to disability and gradually adjusting expectations (referred to as response shift).^54^ Furthermore, sample variation could also be a contributing factor. Each PLWD is unique, and it is possible that some individuals may be more susceptible to functional limitations based on caregiver types, while other outcomes such as depressive symptoms may be influenced by factors not directly associated with caregiver types. The limited availability of longitudinal studies investigating PLWD’s QoL changes in relation to caregiver relationship types underscores the need for further exploration. More research is needed to clarify the role of these QoL outcomes in the context of PLWD and their caregivers.

This study reveals significant interactions between the type of relationship and education attainment in predicting PLWD’s functional limitations. Prior research has not explored on the interaction of education and caregiver type, yet education is consistently linked to health-related factors and behaviors, especially in later life.^55,56^ It is commonly believed that older adults with lower education attainment often correlates with higher likelihood of functional limitations.^57,58^ Our findings suggest that education’s impact on functional declines is associated with the care-recipient relationship type, particularly close kinship (e.g. spouse or children) rather than extended family. Shared caregiving roles might also influence this dynamic. The results might be in part attributable to the participants’ characteristics differences among the groups. Functional limitations in this study were gauged by ADLs assistance, which was reported to differ based on age, marital status, and gender.^58,59^ We observed variations in participants’ demographics among groups, with implications for ADLs assistance. PLWD’s education, as identified in our study, has not previously been recognized in the literature as a predictor of their QoL. It suggests the need for future research on caregiver type effects in the education- QoL association. Acknowledging the role of education and caregiver type on future functional decline will also allow for early identification of older adults with high care needs.

## Limitations and practical implication

Some limitations in our study constrain broad interpretation. First, there is a high rate of loss to follow-up in Round nine (about 45%) in this four-year-period study. This can be attributed to the high death rate of over 35% among the sample. Additionally, the collection procedures set up in the NHATS survey, which sampled individuals residing in nursing homes and residential care, did not complete a sample person (SP) interview and were thus not eligible for a follow-up interview.^26^ Therefore, the data can be considered missing at random. Second, the sample size restricted the number of covariates we were able to use (e.g. caregiver’s co-residence status with the care recipients, care recipients’ multi-morbidity), which may result in a biased estimate of our variable of interest and a possible heterogeneous group.^60,61^ However, we used GEE in data analysis which resides in the unbiased estimation of population-averaged regression coefficients.^62,63^ The use of GEE can give us relatively unbiased estimates on the prediction for how QoL would change by the type of relationship. Future research should have a larger sample and incorporate a broader scope of potential influencing factors to validate and generalize the results of this study. Third, 17.6% proxy respondents were included in this study when older adults could not respond to interviews. The degree of agreement between proxy and self-report depends partly on the domains of QoL being assessed, with observable domains (e.g. assistance in ADLs) having a higher degree of correspondence.^64^ Therefore, the small percentage of proxy respondents is unlikely to have a substantial impact on the QoL outcomes assessed in this study. In addition, an analysis was conducted, and even after excluding the proxy respondents, the findings retained their statistical significance. Fourth, we acknowledged that the measures employed in this study, including PHQ4, general health, functional limitations, and pain, constitute aspects of QoL. However, it is important to note that these measures might not encompass all dimensions comprehensive. Furthermore, it should be noted that the duration and severity of dementia at the time of enrollment, caregiving duration, caregiving hours, and caregiving tasks were not included in this study, which may have affected our results. Future longitudinal studies, including factors such as caregiving outcomes for PLWD, may elucidate the expansion, increased complexity and intensity of the caregiver’s roles and responsibilities in the middle to late stages of caregiving trajectory.^65^

Despite these limitations, our study provides significant evidence about the prediction of care-recipient relationship type on PLWD's QoL change, especially on their functional limitations change. Older adults’ functional limitations reflect their degree of dependence^66^ and are powerful predictors of nursing home admission.^48^ Predicting the functional limitation changes is vital for the healthcare team and policymakers to develop tailored interventions and proactively plan for future healthcare expenses.

## Conclusion

Our study provides significant evidence about the prediction of care recipient relationship type on PLWD’s changes in functional limitations, an important QoL measure. Informal care is integral for developing a sustainable care system for PLWD. Our findings should contribute to raising awareness about the discrepancy in the QoL trajectory of PLWD with different types of caregivers. They provide evidence about the significant contribution of care-recipient relationship type on care recipients’ QoL changes over time. They also help to prioritize resource allocation while addressing the demands for community-dwelling PLWD by socio-demographic characteristics such as education level.

## Supplemental Material


Supplemental Material - Impact of Care-Recipient Relationship Type on Quality of Life in Community-Dwelling Older Adults With Dementia Over Time
Supplemental Material Impact of Care-Recipient Relationship Type on Quality of Life in Community-Dwelling Older Adults With Dementia Over Time by Aiping Lai, Lauren E Griffith, Ayse Kuspinar, Jenna-Smith Turchyn and Julie Richardson in Journal of Geriatric Psychiatry and Neurology.
