# Supplementary material for: Impact of Care-Recipient Relationship Type on Quality of Life in Community-Dwelling Older Adults With Dementia Over Time
Source: J Geriatr Psychiatry Neurol. 2023 Nov 11;37(4):294–306. doi: 10.1177/08919887231215044 (PMC11089829; doi:10.1177/08919887231215044)
Supplement: Supplemental Material - Impact of Care-Recipient Relationship Type on Quality of Life in Community-Dwelling Older Adults With Dementia Over Time [file sj-pdf-1-jgp-10.1177_08919887231215044.pdf]

Appendix I

Care-recipients' quality of life (QoL) outcomes and pertaining questions at the National Health and Aging Trends Study (NHATS)

QoL	Questions at NHATS
Mental Health<br>(scored 0~12, higher score represents worse mental health)	"Over the last month, how often have you<br>(a) had little interest or pleasure in doing things;<br>(b) felt down, depressed, or hopeless;<br>(c) felt nervous, anxious, or on edge;<br>(d) been unable to stop or control worrying".<br>• Each item is scored on a 4- point scale from "not at all" (0), "several days" (1), "more than half the days" (2) to "nearly every day" (3).
General health<br>(scored 0~4, higher score represents worse health)	"Would you say that in general your health is excellent, very good, good, fair, or poor?"<br>• Response is scored on a 5 scale:<br>excellent (0); very good (1); good (2); fair (3); poor (4)
Pain<br>(scored 0~1, 1 represents pain)	"In the last month, have you been bothered by pain?"<br>• Respond "yes" (1) or "no" (0)
Functional Limitations<br>(scored 0~6, higher score represents more severe limitations)	"Whether you received help within the last month with any of the following activities of daily living (ADLs)":<br>(a) eating<br>(b) getting cleaned up<br>(c) using the toilet<br>(d) dressing<br>(e) getting around inside<br>(f) getting out of bed?<br>• Respond "yes" (1) or "no" (0)

Adapted from NHATS data collection instrument form. Available at www. NHATS.org

Appendix II

Older adults' socio-demographic characteristics and dementia status

Variables, Count (%)	Round 5 (n=1230)	Round 6 (n=1137)	Round 7 (n=947)	Round 8 (n=798)	Round 9 (n=673)
**Sex**					
Male	405 (32.9)	372 (32.7)	299 (31.6)	251 (31.5)	211 (31.4)
Female	825 (67.1)	765 (67.3)	648 (68.4)	547 (68.6)	462 (68.7)
**Age**					
65 to 69 yrs	84 (6.8)	60 (5.3)	36 (3.8)	17 (2.1)	3 (.5)
70 to 74 yrs	174 (14.2)	134 (11.8)	112 (11.8)	88 (11.0)	72 (10.7)
75 to 79 yrs	222 (18.1)	190 (16.7)	150 (15.8)	125 (15.7)	107 (15.9)
80 to 84 yrs	272 (22.1)	199 (17.5)	178 (18.8)	154 (19.3)	129 (19.2)
85 to 89 yrs	256 (20.8)	214 (18.8)	175 (18.5)	152 (19.1)	127 (18.9)
90+ yrs	222 (18.1)	208 (18.3)	185 (19.5)	155 (19.4)	148 (22.0)
**Race/ethnicity**					

Non- Hispanic White	735 (60.2)	682 (60.0)	576 (60.8)	481 (60.3)	398 (59.1)
Non- Hispanic Black	386 (31.6)	353 (31.1)	285 (30.1)	246 (30.8)	216 (32.1)
Hispanic	62 (5.1)	59 (5.2)	53 (5.6)	42 (5.3)	34 (5.1)
Other	39 (3.2)	35 (3.1)	27 (2.9)	24 (3.0)	20 (3.0)
**Annual Income**					
<1st quartile (<13000)	305 (24.8)				
1st-2nd quartiles (>=13000 & <22000)	309 (25.1)				
2nd-3rd quartiles (>=22000 & <=40000)	333 (27.1)				
> 3rd quartile (>40000)	283 (23.0)				
**Education**					
Below high school	412 (33.8)				
High school	360 (29.5)				
Above high school below Bachelor	271 (22.2)				
Bachelor and above	177 (14.5)				
**Marital status**					
Married/living with a partner	532 (43.3)	415 (41.4)	336 (40.5)	277 (40.6)	232 (40.0)
Unmarried	698 (56.8)	587 (58.6)	494 (59.5)	405 (59.4)	350 (60.1)
**Living arrangements**					
Alone	249 (20.2)	228 (20.1)	199 (21.0)	169 (21.2)	148 (22.0)
With spouse/partner only	376 (30.6)	283 (24.9)	228 (24.1)	180(22.6)	162 (24.1)
With spouse/partner and others	146 (11.9)	117 (10.3)	97 (10.2)	84 (10.5)	63 (9.4)
With others only	459 (37.3)	374 (32.9)	306 (32.3)	249 (31.2)	209 (31.1)
**Dementia status**					
Probable dementia	371 (30.2)	340 (30.0)	258 (27.3)	194 (24.3)	141 (21.0)
Possible dementia	179 (14.6)	165 (14.5)	126 (13.3)	106 (13.3)	88 (13.1)
No dementia	678 (55.2)	630 (55.5)	562 (59.4)	497 (62.4)	443 (65.9)

% percentage

Appendix III

Comparison between Non-response and Sample in Round 5

Variables, count (percentage)	Non-response (n=557)	Sample in Round 5 (n=1230)
**Age**		
65 to 69 yrs	40 (7.2)	79 (5.7)
70 to 74 yrs	95 (17.1)	137 (11.1)
75 to 79 yrs	92 (16.5)	210 (17.1)
80 to 84 yrs	125 (22.4)	276 (22.4)
85 to 89 yrs	107 (19.2)	275 (22.4)
90+ yrs	98 (17.6)	262 (21.3)
	chi2(5) = 16.1854	P= 0.006
**Dementia status**		

Probable dementia	163 (29.4)	438 (35.6)
Possible dementia	83 (15.0)	187 (15.2)
No dementia	309 (55.7)	604 (49.2)
	chi2(2) = 7.6637	P= 0.022

Appendix IV

Changes in each QoL subscale over 4 years (2015-2019)

Variables, count (percentage)	2015	2016	2017	2018	2019
**Pain**					
Not reporting pain	372 (30.3)	303 (30.3)	280 (33.8)	217 (31.9)	166 (28.6)
Reporting pain	854 (69.7)	696 (69.7)	547 (66.1)	463 (68.1)	415 (71.4)
**PHQ4**					
Low (PHQ4>=0, <=2)	646 (53.7)	523 (53.8)	415 (52.3)	353 (53.4)	309 (54.7)
Symptomized (PHQ4>2, <=12)	556 (46.3)	450(46.3)	378 (47.7)	308 (46.6)	256 (45.3)
**General health**					
Excellent	47 (3.8)	44 (4.4)	35 (4.2)	33 (4.9)	18 (3.1)
Very good	166 (13.5)	160 (16.0)	128 (15.4)	108 (15.9)	91 (15.7)
Good	412 (33.5)	335 (33.4)	279 (33.6)	210 (30.8)	199 (34.3)
Fair	429 (34.9)	337 (33.6)	270 (32.5)	233 (34.2)	196 (33.8)
Poor	175 (14.2)	126 (12.6)	118 (14.2)	97 (14.2)	76 (13.1)
**Functional limitations**					
Not receiving help with any ADL	481 (39.9)	440 (41.0)	356 (40.3)	275 (37.6)	233 (37.2)
Receiving help with 1 ADL	315 (26.1)	203 (18.9)	154 (17.4)	126 (17.2)	99 (15.8)
Receiving help with 2 ADLs	135 (11.2)	119 (11.1)	103 (11.7)	83 (11.4)	77 (12.3)
Receiving help with 3 ADLs	72 (6.0)	68 (6.3)	62 (7.0)	55 (7.5)	44 (7.0)
Receiving help with 4 ADLs	73 (6.1)	56 (5.2)	64 (7.2)	51 (7.0)	39 (6.2)
Receiving help with 5 ADLs	54 (4.5)	82 (7.6)	62 (7.0)	62 (8.5)	57 (9.1)
Receiving help with 6 ADLs	75 (6.2)	105 (9.8)	83 (9.4)	79 (10.8)	77 (12.3)
